# Predictive factors for complications after surgical treatment for schwannomas of the extremities

**DOI:** 10.1186/s12891-019-2538-8

**Published:** 2019-04-11

**Authors:** Toshihide Hirai, Hiroshi Kobayashi, Toru Akiyama, Tomotake Okuma, Hiroyuki Oka, Yusuke Shinoda, Masachika Ikegami, Yusuke Tsuda, Takashi Fukushima, Takahiro Ohki, Yuki Ishibashi, Ryoko Sawada, Takahiro Goto, Sakae Tanaka

**Affiliations:** 1 Department of Orthopaedic Surgery, Faculty of Medicine, The University of Tokyo, 7-3-1 Hongo, Bunkyo-ku, Tokyo, 113-8655 Japan; 2 Department of Orthopaedic Surgery, Saitama Medical Center, Jichi Medical University, 1-847 Amanuma, Omiya-ku, Saitama, 330-8503 Japan; 3 Department of Musculoskeletal Oncology, Tokyo Metropolitan Cancer and Infectious Diseases Center Komagome Hospital, 3-18-22 Honkomagome, Bunkyo-ku, Tokyo, 113-8677 Japan; 4 Department of Medical Research and Management for Musculoskeletal Pain, 22nd Century Medical & Research Center, Faculty of Medicine, The University of Tokyo, 7-3-1 Hongo, Bunkyo-ku, Tokyo, 113-8655 Japan; 5 Department of Rehabilitation Medicine, Faculty of Medicine, The University of Tokyo, 7-3-1 Hongo, Bunkyo-ku, Tokyo, 113-8655 Japan

**Keywords:** Schwannoma, Peripheral nerve, Extremities, Enucleation, Complication

## Abstract

**Background:**

Schwannomas are well-encapsulated, benign neoplasms, and enucleation is a standard operation procedure. The incidence of neurological complications after surgical treatment for schwannomas of the extremities varies, and there is no consensus concerning predictive factors for complications. The aim of this study was to elucidate predictive factors for complications after surgical treatment of schwannomas that develop in the major nerves of the extremities.

**Methods:**

A total of 139 patients with 141 schwannomas arising in major nerves were retrospectively analyzed. Data regarding preoperative clinical features, the postoperative neurological complications, and clinical course of complications, with a median follow-up period of 2 months (range, 0.5–96), were obtained. Predictive factors for complications were statistically analyzed.

**Results:**

Postoperative complications occurred in 49 lesions (34.8%), including 42 with sensory disturbance and 8 with motor weakness. In univariate analysis, older age, tumors originating from the upper extremity, and major motor nerve involvement were associated with a higher complication rate (*p* = 0.03, *p* = 0.003, and *p* = 0.001, respectively). In multivariate analysis, major motor nerve involvement was an independent predictive factor for postoperative complications (*p* = 0.03). Almost all complications gradually improved, but 6 out of 8 patients with motor weakness did not show full recovery at the final follow-up.

**Conclusions:**

Schwannomas originating from major motor nerves can lead to a higher risk for postoperative complications.

## Background

Schwannomas are benign nerve sheath tumors consisting of differentiated Schwann cells. They arise in any site in the body, grow slowly, and are often associated with degenerative changes, including cyst formation, fibrosis, and calcification after bleeding [1]. Over 90% of these lesions are solitary, and multiple schwannomas are a feature of neurofibromatosis type 2.

Resection of the tumor is not always necessary due to its benign nature. When the patient suffers from neurological symptoms, such as spontaneous pain, tenderness, or muscle weakness, surgical treatment is considered. As schwannomas are usually well-encapsulated, except for the rare plexiform variant, enucleation of the tumor with minimum neural damage is the standard surgical procedure [2–5]. However, even with meticulous surgical techniques, neurological complications often occur after surgical treatment for schwannomas. For vestibular schwannomas, microscopic procedures were reported to be useful for reducing neural injury [6]. However, to the best of our knowledge, no study has investigated the utility of the microsurgical technique using a microscope or loupe magnification in the treatment of schwannomas located in the extremities. Whether these operative techniques reduce neurological complications remains uncertain. Thus, the recognition of risk factors for postoperative complications is important, considering the surgical indication for schwannomas.

The incidence of surgical complications reported in previous studies ranges from 1.5–76% [5, 7–15], and the risk factors for postoperative complications are unclear. The discordance concerning the incidence of and risk factors for postoperative complications may be due to differences in study inclusion criteria; inclusion of schwannomas arising in terminal branches within the muscle or skin may have a substantial impact on clinical outcomes. The aim of this study was to identify the predictive factors for complications following surgery for schwannomas, excluding those arising in the terminal branches within the muscle or skin.

## Methods

This was a multicenter, retrospective study that included three institutes. The study design was approved by each institution’s ethical review board, and all patients provided informed consent for treatment and data collection. If the patient was less than 18 years old, informed consent was obtained from the parents or the legal guardians. The study included 139 patients with 141 lesions of the extremities, surgically treated and histologically diagnosed as schwannoma at our institutions from 2009 to 2016. Plexiform schwannomas or schwannomas originating from the terminal branches within the muscle or skin were excluded. Enucleation was performed in all cases: we exposed the capsule of the tumor, made a longitudinal incision into the capsule, and gently peeled it off, avoiding overlying nerve fascicles. The fascicles that entered the tumor were then dissected. A microscope was not used.

Medical records were retrospectively reviewed to obtain data regarding age, preoperative symptoms, previous surgery, tumor location, tumor diameter and the presence of degenerative changes visualized on magnetic resonance imaging (MRI), and postoperative neurological symptoms. Degenerative changes observed on MRI included cyst formation or hemorrhage in the tumor. Postoperative complications were defined as motor weakness and sensory disturbance, including pain, numbness, and hypesthesia observed immediately after operation. Muscular strength was assessed using Medical Research Council (MRC) grading [16]. The clinical courses of the complications were also investigated.

Potential risk factors for postoperative complications were identified by univariate analysis. Statistical analysis was performed using Student’s t-test or Mann-Whitney U-test for continuous variables and chi-square test or Fisher’s exact test for categorical variables. A *P* value < 0.05 was considered statistically significant. Variables identified as significant in univariate analysis were assessed in multivariate analysis. In addition, Bayesian estimation was performed for the variables identified to be significant in the present study, using the results of 5 previous studies and the current results [2, 3, 5, 8, 10]. The previous studies we utilized for Bayesian estimation had the same inclusion criteria and outcomes as ours, or demonstrated all the nerves involved from which we could extract data for the analysis. In the Bayesian estimation, parameter estimates were obtained as precision-weighted averages of the observed data likelihood function and prior distribution. The influence of prior distribution helps stabilize and anchor Bayesian estimates in the presence of small samples [17]. Beta distribution was used for prior distribution in our study. The postoperative complication rates were estimated using the Bayesian Markov chain Monte Carlo approach with the binomial model. To identify significant differences in complication rates, highest posterior density (HPD) intervals were used. Statistical analysis was performed using SAS software (version 9.1.4; SAS Institute Inc., Cary, NC).

## Results

### Baseline characteristics

We included 139 patients with 141 lesions. Baseline characteristic of the patients are shown in Table 1. There were 72 men and 67 women, and the median age of the patients was 56 years (range, 11–84). Two patients underwent surgeries for 2 lesions; one patient had 2 lesions in separate locations, and the other had 2 lesions in the same nerve, and the median duration of postoperative follow-up was 2 months (range, 0.5–96). Preoperative symptoms were observed in 138 lesions (97.8%): numbness was observed in 10 (7.1%), tenderness in 101 (71.6%), radiating pain in 115 (81.6%), hypesthesia in 11 (7.8%), and motor weakness in 6 (4.2%). The median duration of preoperative symptoms was 12 months (range, 1–300). Previous surgery was performed in 3 patients (2.1%). The median tumor diameter was 2.5 cm (range, 1–10). Degenerative changes were seen on MRI in 49 (34.8%) lesions. A tourniquet was used in the operation in 75 (53.2%) lesions. The local distribution of the tumors and the nerves involved are shown in Table 2.

**Table 1:** Baseline characteristics (*N* = 141)

Age, mean (range)	56 (11–84)
Male	72 (51.8%)
Tumor size, median (range) (cm)	2.5 (1–10)
Preoperative symptoms	
Numbness	10 (7.1%)
Tenderness	101 (71.6%)
Radiating pain	115 (81.6%)
Hypesthesia	11 (7.8%)
Motor weakness	6 (4.2%)
Duration of symptoms,	12 (1–300)
median (range) (months)	
Schwannomatosis	12 (8.6%)
Previous surgery	3 (2.1%)
Degenerative changes on MR imaging	49 (34.8%)
Tourniquet	75 (53.2%)

**Table 2:** Tumor locations and complication number (Complication number/Total)

	Number	Locations	Number	Nerves involved	Number
Upper limb	29/60	Upper arm	15/26	Brachial plexus	3/5
		Elbow	5/9	Median	13/21
		Forearm	3/9	Ulnar	5/16
		Hand and wrist	6/16	Radial	5/9
				Musculocutaneous	1/2
				Posterior interosseous	1/1
				Digital	1/6
Lower limb	20/81	Buttock and groin	0/2	Obturator	0/2
		Thigh	3/19	Sciatic	2/7
		Knee	6/17	Femoral	1/3
		Lower leg	8/25	Saphenous	1/7
		Foot and ankle	3/18	Tibial	8/24
				Common peroneal	4/11
				Deep peroneal	1/2
				Superficial peroneal	0/8
				Sural	2/6
				Plantar	1/11

### Predictive factors for postoperative complications

Postoperative complications occurred in 49 (34.8%) lesions. Sensory disturbances, such as pain, numbness, and hypesthesia, were seen in 42 cases (29.8%). Motor weakness developed in 8 cases (5.67%). The original nerves involved in the development of motor weakness were the median nerve in 2 cases, ulnar nerve in 1, radial nerve in 1, posterior interosseous nerve in 1, common peroneal nerve in 2, and tibial nerve in 1.

**Fig. 1:**
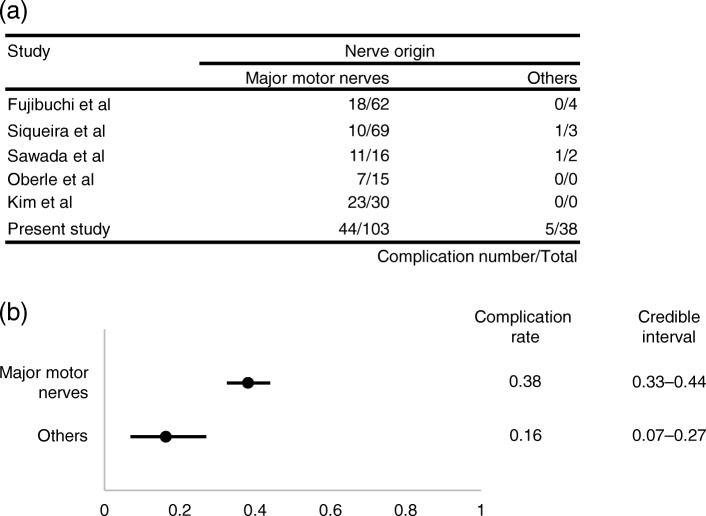
Correlation between nerve origin and postoperative complications. (**a**) Number of complications/total number of cases in previous reports and in the current study. (**b**) Comparison of complication rates between tumors originating from major motor nerves and others

In univariate analysis, shown in Table 3, the following factors were significantly associated with a higher rate of postoperative complications: older age (*p* = 0.03), tumors originating from upper extremity (*p* = 0.003), and tumors originating from major motor nerves (*p* = 0.001). Major motor nerves were defined as nerves including motor bundles, the loss of which affects the performance of daily activities, including the following: brachial plexus, median nerve, ulnar nerve, radial nerve, musculocutaneous nerve, posterior interosseous nerve, obturator nerve, sciatic nerve, femoral nerve, tibial nerve, common peroneal nerve, and deep peroneal nerve. Multivariate analysis demonstrated that tumors originating from major motor nerves was an independent predictive factor for postoperative complications. Bayesian estimation using five previous studies and our data revealed that tumors originating from major motor nerves was a significant predictive factor for postoperative complications; complication rates after surgery (95% HPD intervals) in major motor nerves and in other nerves were 0.38 (0.33–0.44) and 0.16 (0.07–0.27), respectively (Fig. 1).

**Table 3:** Predictive factors for postoperative complications

		Postoperative complications		*P* value	
		(−)	(+)	univariate	multivariate
Mean age		52.2	59.3	0.03	0.06
Tumor size		3.19	2.94	0.45	
Duration of	≥12 months	51	21	0.15	0.25
symptoms	< 12 months	41	28		
Preoperative motor	(−)	89	46	0.41	
weakness	(+)	3	3		
Preoperative hypesthesia	(−)	85	44	0.67	
	(+)	6	5		
Schwannomatosis	(−)	84	45	1.0	
	(+)	8	4		
Previous surgery	(−)	90	48	1.0	
	(+)	2	1		
Degenerative changes	(−)	59	31	0.9	
on MR imaging	(+)	32	17		
Tourniquet	(−)	42	24	0.25	
	(+)	50	25		
Location	Upper limb	31	29	0.003	0.14
	Lower limb	61	20		
	Proximal	46	27	0.59	
	Distal	46	22		
Nerve	Major motor	59	44	0.001	0.03
	Others	33	5

### Clinical course of the postoperative complications

The median postoperative observation period was 2 months (range, 0.5–96). Improvement in new-onset sensory symptoms such as pain, numbness, and hypesthesia were observed in 41 out of 42 patients. Medications including gabapentin, pregabalin, or tramadol were prescribed for 9 patients, and were discontinued within 1 year in all of the patients. Muscle power improved to some degree in all 8 patients with postoperative motor weakness, but 6 out of the 8 patients did not show recovery to preoperative levels at the final follow-up (Table 4).

**Table 4:** Clinical course of motor weakness due to surgery

		MRC grading			Postoperative observation
Case	Nerves involved	Preoperative	Postoperative	Final follow-up	period (months)
1	Median	5	4	5	48
2	Ulnar	5	3	4	41
3	Median	5	2	3	2
4	Radial	5	2	4	1
5	Posterior interosseous	4	2	4	96
6	Common peroneal	5	2	3	21
7	Common peroneal	4	0–1	2	16
8	Tibial	5	0–1	2	6

*MRC* Medical Research Council

## Discussion

Previous studies reported that surgery for schwannomas originating from unidentified terminal branches in the muscle or skin did not cause postoperative neurological symptoms [18]. We evaluated the predictive factors for postoperative complications of schwannomas involving major nerves. Although several risk factors have been reported, there is no consensus regarding the importance of these factors. The current analysis includes the largest number of patients among studies investigating risk factors for postoperative complications in schwannomas of the extremities thus far. We identified tumors originating from major motor nerves as an independent predictive factor. This was confirmed by Bayesian estimation, integrating results from 5 previous studies.

Several previous reports stated that the incidence of postoperative complications was significantly higher in younger patients [3], patients with larger tumors [3–5], tumors with a proximal location, and tumors originating from the ulnar nerve [10], but these features were not identified as risk factors in this study. Abe et al. reported that small tumors with numbness significantly correlated with postoperative neurological deficits [15], which has not been identified as a risk factor in other studies. Our study demonstrated that the involvement of nerves with motor function affecting the quality of daily activities was an independent risk factor for complications, even when integrating previous reports by Bayesian estimation. The incidence of complications in schwannomas originating from major motor nerves was 42.7% in the present study. Kim et al. reported that 76.7% of patients with schwannomas originating from major motor nerves of the lower extremities developed immediate postoperative neurological deficits [5]. This is the highest complication rate reported so far and might support our results.

Regarding the clinical course of postoperative neurological complications, several studies reported that both sensory and motor deficits improved to some degree in 73–100% of patients [3–5, 15]. They unanimously showed that most sensory disturbances caused by operation subsequently improved to full recovery or mild disorders with no need for medication or nerve block. On the other hand, many preceding studies reported that postoperative muscle weakness was not fully recovered [3, 5, 8, 15]. Similar results were obtained in our study. This suggests that surgeons should pay greater attention to schwannomas originating from a major motor nerve.

The present study has some limitations. First, this is a retrospective study. Due to the benign nature of these tumors, the surgical indication for schwannomas differs not only among patients but also among surgeons. In our study, surgery was performed by multiple physicians. The follow-up periods were relatively short and differed among patients. The residual neurological deficits may further improve with long observation periods. Second, intraoperative findings were not fully investigated. Data about the appearance of fascicles over the capsule of the tumor and the presence of residual tumor after operation is lacking. Abe et al. reported that the incidence of complications is higher in schwannomas with many fascicles widely splayed over the tumor capsule than in those with a small number of fascicles [15].

## Conclusions

Our analysis suggests that schwannomas originating from nerves with major motor function are associated with greater risk of postoperative neurological complications. Data collected in this study will serve to help clinicians inform patients about the risk of postoperative complications.

## Abbreviations

HPD: highest posterior density
MRC: Medical Research Council
MRI: magnetic resonance imaging
